# Cascade Release Nanocarriers for the Triple-Negative Breast Cancer Near-Infrared Imaging and Photothermal-Chemo Synergistic Therapy

**DOI:** 10.3389/fonc.2021.747608

**Published:** 2021-09-15

**Authors:** Ke Li, Ruyue Li, Baona Zhou, Jing Chen, Kai Lan, Wenhua Zhan, Di Chen, Tao Zhang, Xueping Li

**Affiliations:** ^1^Institute of Basic and Translational Medicine, Xi’an Medical University, Xi’an, China; ^2^College of Clinical Medicine, Xi’an Medical University, Xi’an, China; ^3^Department of Radiation Oncology, General Hospital of Ningxia Medical University, Yinchuan, China; ^4^Department of Basic Medical Science, Xi’an Medical University, Xi’an, China; ^5^Ultrasonography Department, Xingping Maternity and Child Health Hospital, Xingping, China

**Keywords:** triple-negative breast cancer, nanocarriers, triggered release, synergistic therapy, near-infrared imaging

## Abstract

Triple-negative breast cancer (TNBC) has inadequate treatment approaches and a poor prognosis. It is urgent to develop new treatment approaches for TNBC. The combination of photothermal therapy (PTT) and chemotherapy is a very effective potential therapy for TNBC. However, asynchronous accumulation, unclear efficacy, and toxic side effects hinder the further promotion of this method. Therefore, we designed and constructed a new type of nanocarriers, the cascade release near-infrared imaging (NIFI) & thermal-chemo combination nanoparticles (CNC NPs), that can release drugs through the cascade of ultrasound triggering and pH responding to achieve the synchronous tumor accumulation, monitoring and synergistic treatment of two functional molecules. The key material of CNC NPs is the polydopamine (PDA), which, through self-assembling, forms a rigid shell that contains doxorubicin (DOX) and NIF fluorescent dye IR780 on the surface of the perfluorohexane (PFH) microbubbles. The results show that CNC NPs have a hollow core-shell structure with an average particle size of 97.3 ± 27.2 nm and have exceptional colloidal stability and photothermal conversion efficiency. The NPs can effectively perform cascade drug release through ultrasound triggering and pH responding. CNC NPs have good *in vivo* biological safety and excellent fluorescence imaging, drug delivery, and therapeutic abilities in the TNBC models. These results provide an experimental basis for the development of new clinical treatment methods for TNBC.

## Introduction

TNBC is a special high-risk subtype of breast cancer. It is difficult to be treated by endocrine therapy and targeted therapy due to its lack of corresponding targets. At present, traditional surgery, radiotherapy, and chemotherapy are still the mainstays for TNBC treatments. However, the prognosis of TNBC is far worse than that of the other types of breast cancer (1–4). There is an urgent need to develop new specific TNBC treatment methods. PTT converts light of a specific spectrum into thermal energy to ablate tumor tissues, and has great advantages such as minimal invasiveness, high specificity, and fewer side effects (5, 6). The essence of tumor PTT is the photothermal conversion agents. Materials used for photothermal conversion include inorganic metal materials, carbon nanomaterials, porphyrin derivatives, and NIF fluorescent dyes. Compared with other materials, NIF fluorescent dyes, which have been applied in clinical (7–12), have the advantages of high photothermal conversion efficiency, insignificant toxic and side effect, high metabolizability, and ideal absorption spectrum. In addition, NIF dye can also perform function of imaging probe. However, during the PTT, the heterogeneity of the tumor tissue will cause uneven heat distribution inside the tumor, leading to incomplete treatment (13). Therefore, other therapeutic technologies are needed to combine with PTT for supplement of disadvantage. At present, a variety of tumor treatment methods combined with PTT have been explored and developed (14–16). Among them, the PTT and chemotherapy combination has better prospects than those of the other combined methods. The thermal effect produced by PTT can directly ablate tumor tissues and help chemotherapeutic molecules better enter the tumor cells and further intervene the remaining tumor cells, exhibiting a good synergistic effect (17–19).

Although traditional combination therapy has obvious advantages, its current results are not satisfactory (20, 21). The essential prerequisite for the combined PTT and chemotherapy is that the two therapeutic molecules simultaneously accumulate in the tumor. However, there are considerable differences in the metabolic pathways of the different types of molecules. Moreover, the inherent biological complexity and dynamic changes of tumors make it difficult for therapeutic molecules to accumulate in tumor tissues simultaneously, which is significantly weakening the synergy (22, 23). Therefore, how to achieve synchronous delivery becomes the main problem of current combined tumor therapy.

Functional nano-drug delivery systems have opened up new gate for solving the problem of combined therapy. The most common way for nanocarriers to accumulate in the tumor is through the enhanced permeability retention effect (EPR) of solid tumors (24). However, this way still has defects such as non-positive targeted aggregation and side effects (25). Stimulus-responsive nanocarriers greatly improved the drug delivery performance of nanocarrier *in vivo*. Such carriers can change their structure and shape under external stimuli such as pH, temperature, ultrasound waves, and light to achieve controlled drug release (26, 27). The acidic nature of the tumor microenvironment allows many nanocarriers to release the therapeutic molecules by the pH response, realizing the targeted delivery (28). However, pH responding release is a slow and sustained process. The release rate and efficiency are low and uncertain. Therefore, together with a more quickly release method, it can get better tumor accumulation. Ultrasound is entering the view of researchers. The technology has been widely used in clinical diagnosis and imaging (29). In research of drug delivery, ultrasound has been reported that can help drugs permeate various physiological barriers. And some ultrasound phase change molecules make the method be a good source of external stimulus (30). The perfluorocarbon is a popular ultrasound triggering material, which occurs a phase change under the ultrasound, causing the carrier to rupture and release the loaded drug (31). However, perfluorocarbon is normally in a liquid state and must be encapsulated in microbubbles by phospholipids, polymers, and surfactants. There are still many technical problems for perfluorocarbon to be stably wrapped in nanoparticles (32, 33).

In order to solve this problem, after referring to a variety of preparation protocols (34–37), PDA was chosen to prepare a composite nanocarrier with a rigid shell. PDA composite nanocarrier can load drugs and ensure the perfluorocarbon’s long-term stability, achieving cascaded release in response to ultrasound and pH stimulus. The monomer of PDA is dopamine, a small molecule with a benzene ring, which can be polymerized by π-π stacking and hydrogen bonding under alkaline conditions, forming a strong PDA compound on the surface of other materials. On the other hand, the PDA layer can be gradually decomposed in the acidic environment (38–40). During PDA polymerization, molecules that also have benzene rings can be encapsulated in PDA. This feature can be used for drug loading (41, 42). Both the chemotherapeutic drug DOX and the NIF dye IR780 have benzene rings. The encapsulation of these two molecules in PDA has been reported in many publications (37, 41). Based on the facts mentioned, we developed a multifunctional nanocarrier, CNC NPs, with PDA as the coating material, PFH as the vacuolating agent, DOX as chemotherapeutic molecule, and IR780 as the photothermal conversion agent and NIFI agent. After entering the tumor tissue, CNC NPs achieve their first-stage release under ultrasound and then further release through the low pH microenvironment of the tumor to achieve the effect of NIFI and synergistic therapy. The nanocarrier delivery and therapeutic effects were evaluated by the *in vitro* and *in vivo* TNBC models. CNC NPs are expected to provide an experimental basis for developing new clinical treatment methods for TNBC.

## Materials and Methods

### Materials

Dopamine hydrochloride, doxorubicin hydrochloride, IR780, perfluorohexane (PFH), and coumarin-6 were all purchased from Aladdin Biochemical Technology Co., Ltd. PFH special surfactant (FS63) was purchased from Guangzhou Jieluhua Co., Ltd. CCK -8 kit, DAPI kit, apoptosis detection kit, interleukin-6 (IL-6) detection kit, and tumor necrosis factor-α (TNF-α) detection kit were purchased from Beyotime Biotechnology Co., Ltd. The other chemical reagents were purchased from Sinopharm Group. The MCF-7, MCF-10A, and MDA-MB-231 cell lines were all derived from ATCC. BALB/c mice and BALB/c-nu/nu mice were purchased from Beijing HFK Biotechnology Co., Ltd. Unless otherwise stated, all the chemicals and reagents were of analytical grade and used as received.

### Preparation of CNC NPs

CNC NPs were prepared according to Zhu et al.’s publication (37). In brief, 100 μL PFH and 120 μL FS63 were mixed and dispersed in 3 mL Tris solution (50 mmol/L). The mixture solution was continuously stirred at high speed during the dispersion. After that, 100 μL IR780 solution (10 mg/mL, DMSO) and 100 μL DOX solution (10 mg/mL) was added to the solution. Then the solution was sonicated with 150 W power for 5 min. Finally, 1 mL dopamine hydrochloride solution (10 mg/mL) and 100 μL H_2_O_2_ (3%, v/v) were added. The solution was sealed and rotated in the dark for 48 h. After that, the final products were obtained through dialysis, removing DMSO and other soluble impurities. A Malvern instrument (NS-90, Malvern, UK) was used to investigate the obtained CNC NPs preliminarily.

### Properties Characterization of CNC NPs

The morphology of CNC NPs was characterized by a transmission electron microscope (TEM). CNC NPs was decomposed by acid liquor, and then dehydrated by lyophilizer (FD-1A-50, LANYI, Shanghai, China). The concentration of DOX and IR780 in the lyophilized CNC NPs residue was detected by a fluorescence spectrophotometer, and the encapsulation rate and drug loading of the two compounds were calculated. The CNC NPs were dispersed in PBS, complete medium, and fetal calf serum (FBS) to simulate different physiological environments. The colloidal stability was evaluated by particle size changes. An 808 nm laser was used to irradiate CNC NPs in different concentrations, irradiation power, and conditions to evaluate the photothermal conversion efficiency. *In vivo* evaluation of thermal conversion was performed in BALB/c mouse model. The CNC NPs was subcutaneously injected in the crotch of mouse. Subsequently, the 808 nm laser was used to irradiate the injection area. The infrared thermal imager (E4, FLIR, US) was employed to measure the temperature of the experiment. The release degree of CNC NPs under different pH and temperature conditions was detected by the dialysis method. The response release of CNC NPs was evaluated using an ultrasound system (Voluson E8, GE, USA).

### CNC NPs Cell Suppression

The cytotoxicity of CNC NPs was preliminarily evaluated by the CCK-8 assay. The cell lines were MCF-7, MCF-10A, and MDA-MB-231. The cells were seeded in a 96-well plate (1×10^4^ cells/well) and incubated at 37°C and 5% CO_2_ for 24 h. CNC NPs, empty NPs, DOX, and IR780 were added into the wells, respectively. The cells were incubated for another 72 h before the CCK-8 solution was added. After incubating with CCK-8 for 2 h, the cells were put into a microplate reader (ELx800, BioTek, USA) to test the 450 nm absorbance for calculating the cell viability.

The combined treatment of PTT-chemotherapy was evaluated by CCK-8 assay, colony formation test, and flow cytometry. The cell line was MDA-MB-231. The CCK-8 assay was carried out similarly to the method mentioned above except that the cells concentration was 2×10^4^ cells/well. The cells were irradiated with an 808 nm laser after adding the samples and then incubated for 24 h. In the colony formation test, the cell suspension was added into a 1.5 mL centrifuge tube at 2000 cells/mL. The sample was added, and laser irradiation was performed, and then the cell suspension was transferred to a dish and cultured for 5 d. After that, the colony was stained for observation. After the cells are treated with PTT, chemotherapy, and combination therapy, the cells were stained with a cell apoptosis kit and tested by a flow cytometer (Accuri C6 Plus, BD, USA).

### CNC NPs Cell Delivery

Firstly, besides DOX’s red fluorescence, coumarin-6 was used to label CNC NPs with green fluorescence. The MDA-MB-231 cells were seeded at 2 × 10^5^/plate in a confocal microscopy dish. The cells were cultured at 37 °C and 5% CO_2_ for 24 h before being added with fluorescent-labeled NPs for further incubation. The dishes at different time points were fixed and labeled with DAPI. The cells were observed with a laser confocal microscopy (TCS SPT, Leica, Germany). In order to determine the internalization effect of CNC NPs, the cells were pre-treated with sodium azide (NaN_3_), an endocytosis inhibitor, and incubated with CNC NPs for confocal observation and comparison.

### *In Vivo* Toxicity of CNC NPs

A hemolysis test was performed to evaluate CNC NPs’ effect on red blood cells. 2% erythrocyte suspension was prepared and divided into groups. Then, CNC NPs, IR780, DOX, PFH+FS63 and PDA were added to the test groups, respectively, while the water, saline, and 0.1% Triton X-100 were added to the control groups, respectively. The erythrocytes were incubated at 37 °C for 2 h. Because CNC NPs, PDA, DOX and IR780 have color, which could interfere absorbance of the sample. After a centrifugation, the supernatant was replaced by the same amount of water. Then, the precipitate was mixed and incubated for another 4 h. The absorbance wavelength at 540 nm in each group was measured by a microplate reader, and the hemolysis rate was calculated.

In the acute toxicity test, 50 BALB/c mice, half male and half female, weighing about 20 g, were selected and randomly divided into 5 groups. CNC NPs, PFH + FS63, IR780, DOX, and PDA were administrated into the mice through the intravenous injection, respectively. The mice were observed continuously for 14 d. The symptom and sign were recorded, and the survival rate was calculated. Finally, the surviving mice were euthanized. The heart, liver and kidney were collected for pathological analysis.

The levels of IL-6 and TNF-α in the blood of mice were tested by the ELISA kit to evaluate whether CNC NPs can cause a systemic inflammatory reaction *in vivo*. The samples were injected through the tail vein, and the blood samples were collected 24 h after injection.

All animal experiments in this work were authorized by the Laboratory Animal Administration Committee of Xi’an Medical University. The protocols for animal experiments followed the Guidelines for the Use and Care of Experimental Animals at Xi’an Medical University. The Animal Ethics Approved Document Number is XY-AUC-2020-352.

### *In Vivo* Tumor Xenograft Model

4-week-old female BALB/c-nu/nu mice were reared in an SPF room for 5 d to adapt to the environment. Then 150 μL MDA-MB-231 cell suspension (1×10^6^/mL) was injected into the crotch of each mouse. The tumor-bearing mice were used for subsequent experiments after their tumors grew to a suitable volume.

### *In Vivo* NIF Imaging and Triggered-Release of CNC NPs

Two single side tumor-bearing mice were injected with CNC NPs and the same concentration of IR780 solution, respectively. The distribution of fluorescent signals in mice was continuously observed by the IVIS imaging system (PE, USA). The excitation wavelength was 780 nm, and the emission wavelength was 845 nm. After the observation, the mice were euthanized, and the main organs and tumor tissues were taken out to analyze the distribution of CNC NPs *in vivo*.

Two double side tumor-bearing mice were injected with CNC NPs and the same concentration of IR780 solution, respectively. After injection, the right-side tumor was treated by ultrasound probe (11L-D, voluson E8, GE, USA) for 20 min. The imaging was performed to observe the effect of ultrasound triggering *in vivo*.

### Antitumor Effect of CNC NPs *In Vivo*

Thirty-five single side tumor-bearing mice were divided into seven groups. The treatment methods were saline, IR780 + laser, DOX, IR780+DOX+laser, IR780+DOX+triggered+laser, CNC NPs+laser, and CNC NPs+triggered+laser. The administration was through intravenous injection. Ultrasound irradiation was carried out 1 h after the administration for six cycles of 5 min treatment and 3 min interval. Two hours after ultrasound treatment, the laser irradiation was performed with the irradiation wavelength of 808 nm. The irradiation power was 1 W/cm^2^ and the irradiation time was 3 min. The whole intervention process was conducted twice a week. The size of tumors and the weight of mice were measured continually, and the tumor area was photographed. After 21 d, the mice were euthanized, and the tumor tissues were taken, weighed, and photographed.

### Statistical Analysis

Two ways ANOVA and t-test were used for statistical analysis. The software was GraphPad prism 5.0. A P-value less than 0.05 indicated a statistical difference. The data of independently repeated experiments were presented as the mean values ± standard deviation (SD). The statistical differences between the groups were indicated p<0.05.

## Results

### Characteristics and Performance of CNC NPs

**Figure 1:**
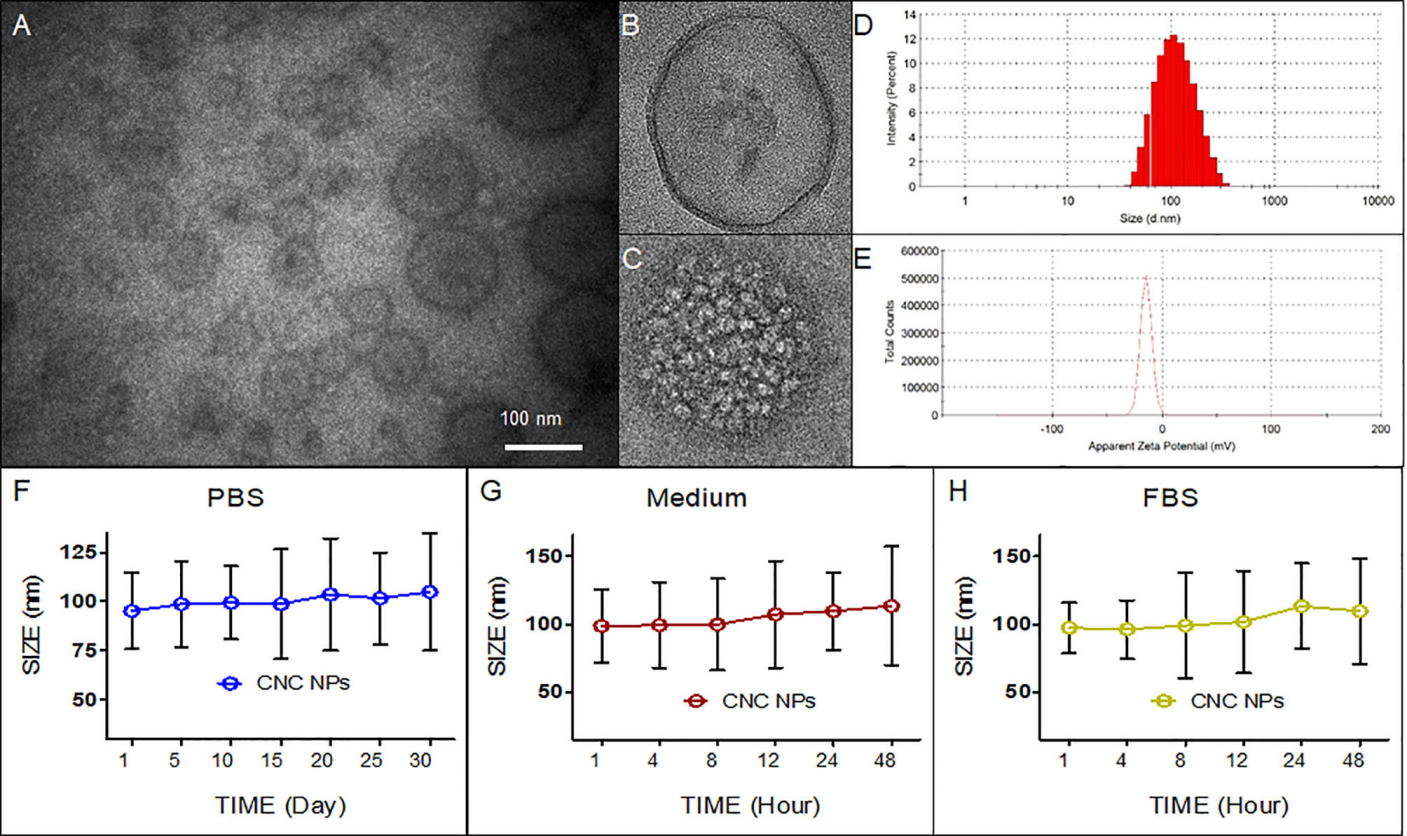
Characteristics of CNC NPs. The morphology **(A–C)**, size distribution **(D)**, zeta-potential distribution **(E)**, and colloid stability test results **(F–H)**.

Firstly, the morphology of CNS NPs was observed by TEM. **Figure 1A** shows that the CNC NPs have spherical shape, mono-dispersity, and hollow-shell structures. **Figures 1B, C** show the details of empty NPs and CNC NPs, respectively. With the same amount of FS63 surfactant, the PFH concentration changes led to different numbers of internal cavity. There is obvious cavitation inside of CNC NPs, due to PFH is vaporized in sample preparation of TEM. The particle size of CNC NPs was 97.3 ± 27.2 nm with a wide distribution range (**Figure 1D**). Since the inner core of CNC NPs is droplet micelle, it is difficult to control their size. The overall particle size meets the requirements of effectively entering the tumor tissue through the EPR effect. The results show that the structure of the CNC NPs meets our expectation. **Figure 1E** shows that the surface potential of CNC NPs was approximately -14 mV, which indicates that the CNC NPs can maintain a good circulation *in vivo*. The stability test results are shown in **Figures 1F–H**. During the experiment, the particle size of CNC NPs did not change significantly in different dispersion environments, showing excellent colloidal stability. The encapsulation rate and drug loading of DOX by CNC NPs were 90.3 ± 5.5% and 12.6 ± 3.2%, respectively. And the encapsulation rate and drug loading of IR780 by CNC NPs were 94.6 ± 3.1% and 13.8 ± 1.3%, respectively.

**Figure 2:**
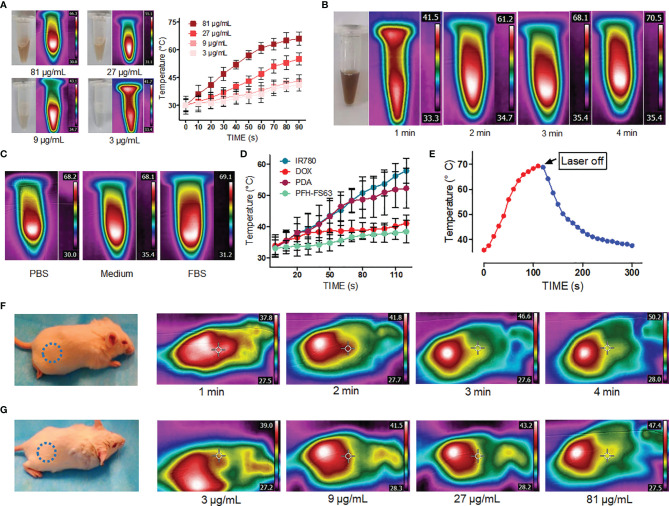
The photothermal properties of CNC NPs. The heating -rate effect of the CNC NPs, with different concentrations **(A)**, under different irradiation time duration **(B)**, in different solution environments **(C)**. The temperature elevation of each component of CNC NPs **(D)**. The heating-cooling curve of CNC NPs **(E)**. The *in vivo* temperature elevation of the CNC NPs under different time points. The blue dotted circles indicate the areas of injection **(F)**. The *in vivo* temperature elevation of the CNC NPs under different concentrations. The blue dotted circles indicate the areas of injection. **(G)**. The quantitative experiment was repeated three times.

**Figure 2** shows the evaluation results of photothermal conversion of CNC NPs. It can be seen from **Figure 2A** that, under the same irradiation power and time, there is a positive relationship between the concentration of CNC NPs and temperature. With a concentration of 81 μg/mL, the highest temperature was 66.3 °C. The temperatures of the other concentrations are all higher than 41 °C. **Figure 2B** shows the heating process of the sample under different irradiation time-lengths. The temperature rose obviously with the extension of irradiation time, reaching 69 °C in about 4 min. The temperature rising in different solvents is shown in **Figure 2C**. The solvent has no apparent influence on the photothermal conversion of CNC NPs. The photothermal conversion effects of different components of CNC NPs were measured (**Figure 2D**). The result shows that IR780 was the primary substance causing the temperature to rise, followed by PDA. **Figure 2E** is the temperature changing curve of CNC NPs. With laser irradiation, the temperature rose rapidly. While after the irradiation, the temperature dropped slowly to room temperature. The *in vivo* evaluation of CNC NPs is illustrated in **Figures 2F, G**. In **Figure 2F**, the temperature rose in the injected area obviously with the extension of irradiation time, reaching 49 °C in about 4 min. In **Figure 2G**, after 2 min of irradiation, the temperature rose also exhibited a positive relationship with concentration of CNC NPs. These phenomena indicated that skin could not obstruct NIF absorption of CNC NPs. The results show that CNC NPs have an excellent photothermal conversion effect.

**Figure 3:**
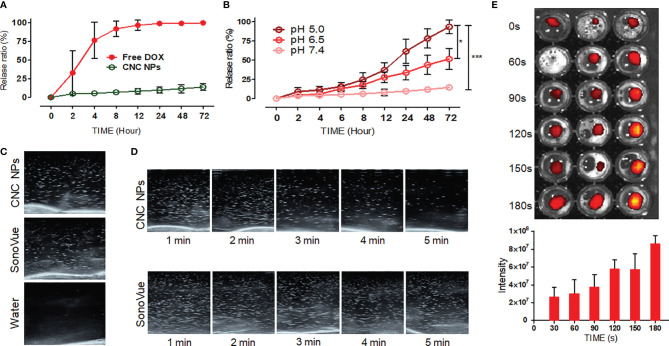
The releasing evaluation of CNC NPs. The DOX releasing curve of CNC NPs **(A)**. The releasing results of CNC NPs in different pH environments **(B)**. The ultrasonic cavitation effect of CNC NPs, the commercialized ultrasonic imaging agent, SonoVue, as the control **(C)**. The cavitation time duration results of CNC NPs **(D)**. The ultrasound triggered release results of CNC NPs **(E)**. The quantitative experiment was repeated three times. The “***” and “*” symbols represent P values less than 0.001 and 0.05, respectively.

In the design, CNC NPs should be stable without stimuli and release drugs rapidly after triggering. The triggered release results are shown in **Figure 3**. **Figure 3A** shows the DOX release curve of free DOX and CNC NPs. The result shows that most of the free DOX was released in the first 4 h, while the CNC NPs almost did not release any DOX in 72 h. **Figure 3B** is the release curve of CNC NPs in different pH environments. The DOX release rate gradually increased with the decrease of pH, indicating that CNC NPs can effectively release the payload in the acidic environment. **Figures 3C, D** are the ultrasonic images of CNC NPs. In the figures, under ultrasound treatment, CNC NPs have the same cavitation signal as SonoVue, a commercialized ultrasonic contrast agent. There is no such signal in pure water. With the extension of time, the ultrasound signal of CNC NPs gradually faded and almost disappeared in about 5 min, indicating that CNC NPs have a rapid triggered release effect. The outer layer of CNC NPs is PDA, which was a kind of melanin and had robust light absorption capability. **Figure 3E** further proves the ultrasound triggered release of CNC NPs. With the extension of triggering time, IR780 was constantly released from CNC NPs, and its fluorescence intensity was enhanced. These results indicate that CNC NPs have a good response and release ability.

### *In Vitro* Cytotoxicity of CNC NPs

**Figure 4:**
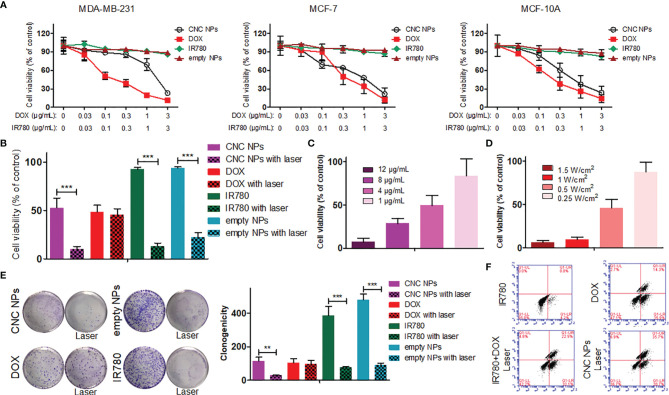
The *in vitro* cytotoxicity of CNC NPs. The results of CCK-8 assay in MDA-MB-231, MCF-7 and MCF-10A cell lines under treatment of CNC NPs and its main components **(A)**. The comparison of cell survival ratios between treatment and non-treatment by laser irradiation in CNC NPs and its components **(B)**. The *in vitro* PTT effect of CNC NPs with different concentrations **(C)** and under different power irradiations **(D)**. The comparison of colony formation between treatment and non-treatment by laser irradiation in CNC NPs and its components **(E)**. The flow cytometry results of MDA-MB-231 cell line treated with chemotherapy, PTT, combination treatment and CNC NPs **(F)**. Each quantitative sample has three duplications. The “***” and “**” symbols represent P values less than 0.001 and 0.01, respectively.

In this study, a breast epithelial cell line (MCF-10A), a breast cancer cell line (MCF-7) and a TNBC cell line (MDA-MB-231) were used to detect the inhibition effect of CNC NPs *in vitro*. As shown in **Figure 4A**, the empty NPs and IR780 did not show significant cytotoxicity at all the concentrations, while the toxicity of DOX rose with the increase of concentration. CNC NPs showed a similar inhibitory effect as that of the DOX group, indicating that CNC NPs can effectively deliver drugs into cells and exert an inhibitory effect. No significant difference of CNC NPs’ toxicity was found between the normal cell and breast cancer cell lines.

After adding PTT, the results were much different. As **Figure 4B** shown, CNC NPs had a noticeable cell ablation effect after receiving laser irradiation, and the cell survival rate decreased to less than 10%. The empty NPs also showed a certain cell ablation effect. **Figures 4C, D** show that the cell survival rate has a significant positive relationship with the time and intensity of laser irradiation. The colony formation assay results (**Figure 4E**) further prove the inhibitory effect of CNC NPs. Flow cytometry results (**Figure 4F**) show that CNC NPs could effectively induce apoptosis, which was even more obvious than chemotherapy or PTT alone.

### CNC NPs Cell Delivery

**Figure 5:**
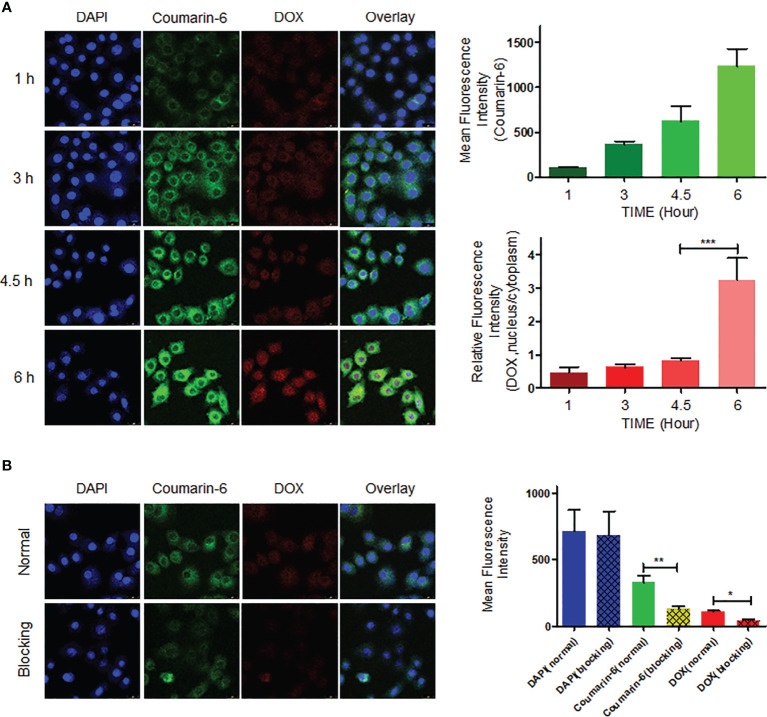
The cell endocytosis test results. The intracellular accumulation of fluorescent signal and fluorescence quantitative analytics results of CNC NPs **(A)**. The endocytosis inhibition and fluorescence quantitative analytics results of CNC NPs **(B)**. The data of signal quantitation were extracted at more than three areas. The “***“, “**“ and “*” symbols represent P values less than 0.001, 0.01 and 0.05, respectively.

MDA-MB-231 cells were incubated with fluorescence-labeled CNC NPs and observed at different time points. It can be seen from the results (**Figure 5A**) that the intracellular fluorescent signal gradually edged up over time and reached its peak at 6 h. In addition, the distribution of the fluorescent signal also changed significantly. Coumarin-6’s green fluorescence always remained in the cytoplasm. Because of its lipophilicity, coumarin-6 mainly accumulated in various membrane structures in the cell membrane and cytoplasm. Red fluorescent DOX mainly combines with DNA, gradually accumulating in the nucleus. As shown in the figure, the red fluorescent signal was mainly in the cytoplasm before 4.5 h. After that, the red fluorescent signal in the nucleus rose gradually, indicating that CNC NPs can effectively deliver drug molecules into cells and gradually release them. **Figure 5B** is the result of endocytosis blocking teat. The fluorescent signals in the cells that pretreated by NaN_3_, were significantly lower than those in the cells without the pretreatment, indicating that CNC NPs enter into the cells through endocytosis.

### Toxicity of CNC NPs *In Vivo*

**Figure 6:**
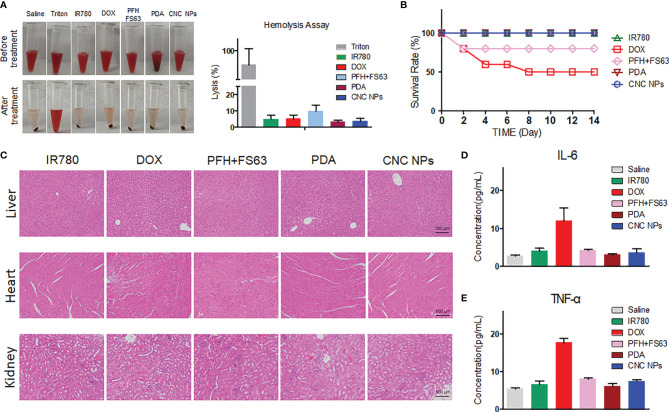
*In vivo* toxicity evaluation of CNC NPs. The hemolysis test results of CNC NPs and its main components. The tubes in the top row are the mixtures of red cell suspension and samples, and the tubes in the bottom row are the mixtures after incubation and centrifugation **(A)**. The *in vivo* acute toxicity test results of CNC NPs on the BALB/c mice **(B)**. The pathological analysis of CNC NPs and its main components to the heart, liver, and kidney **(C)**. The effects of CNC NPs and its components to the main inflammatory factors *in vivo* **(D, E)**. Each quantitative sample has three duplications.

*In vivo* safety is crucial to the application of CNC NPs. Firstly, the effect of CNC NPs on red blood cells was evaluated by a hemolysis test. The results are shown in **Figure 6A**. From the hemolysis rate, as the positive control, the Triton X-100 caused severe hemolysis that more than 85% of the red blood cells were damaged. The hemolysis rates of other treatment groups were relatively low. No significant hemolysis occurred in either CNC NPs or their component, indicating their good injection safety. After that, the acute toxicity of CNC NPs to BALB/c mice was evaluated. The survival rate curve of mice is shown in **Figure 6B**. Two mice in PFH+FS63 treatment group died on the day of injection. Five mice in the DOX group died in succession. The other mice were still alive on the 14th day. The pathological analysis results are shown in **Figure 6C**. In the DOX group, the heart tissue had apparent damage. In IR780 and CNC NPs groups, some inflammatory cells infiltration and a few binuclear and megakaryocytes appeared in the liver tissue. No noticeable pathological change was found in the kidney. The results preliminarily prove the safety of CNC NPs *in vivo*. The concentrations of IL-6 and TNF-α also further prove that CNC NPs would not cause a significant systemic inflammatory response (**Figures 6D, E**). The concentrations of IL-6 and TNF-α in the DOX group were much higher than those in the other groups, but the concentrations of the two factors in the CNC NPs group remained low, similar to those of the normal saline group. These results show that CNC NPs have good biocompatibility *in vivo*.

### *In Vivo* NIF Imaging and Triggered-Release of CNC NPs

**Figure 7:**
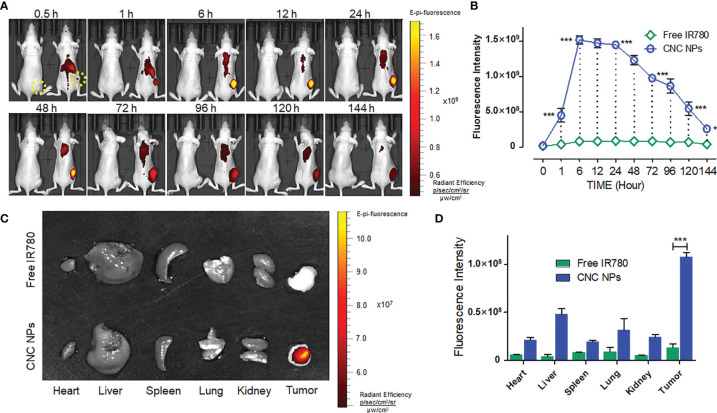
*The in vivo* NIF imaging of CNC NPs. The dynamic monitoring of CNC NPs in the TNBC model mice and the fluorescent signals quantitative results of the tumor region. The yellow dotted circles indicate the areas of tumors. **(A, B)**. The residual fluorescent signals and their quantitative results in the organs and tumors of the mice **(C, D)**. The data of signal quantitation were extracted at more than three areas. The “***” and “*” symbols represent P values less than 0.001 and 0.05, respectively.

The *in vivo* NIFI observation of CNC NPs is shown in **Figure 7**. It can be seen from **Figures 7A, B** that the fluorescent signals in the CNC NPs injected mice were significantly higher than those in the equivalent IR780 injected mouse. In the CNC NPs injected mouse, the fluorescent signals first gathered in the liver and spleen and then appeared in the tumor and lung. The signals reached their high intensities at 6 h and maintained those intensities until 48 h before they decreased significantly. It is worth noting that the signals in tumors had been maintained until 144 h, showing excellent *in vivo* long circulation and monitoring ability. The results of residual fluorescence detection in the organs and tumors are shown in **Figures 7C, D**. In the CNC NPs injected mice, fluorescent signal still retained in the tumor area one week after the injection but did not gather much in the organs. In contrast, the IR780 injected mouse have weaker fluorescent signals than those of the CNC NPs injected mouse. The results show that CNC NPs can realize long-term drug circulation *in vivo* and have a certain tumor monitoring ability.

**Figure 8:**
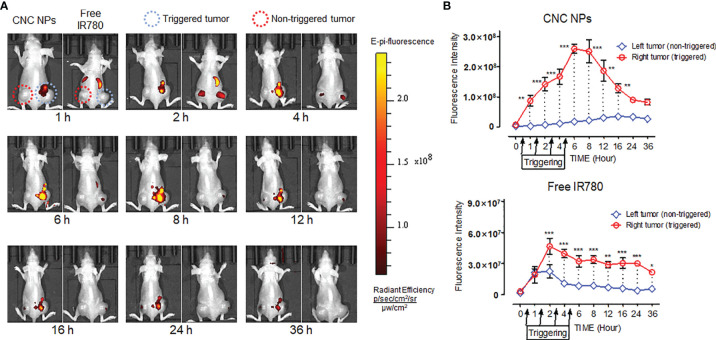
The *in vivo* ultrasound triggered release results of CNC NPs. The models were hepatic carcinoma double-tumor mice. The ultrasound probe was only applied on the right tumor of each mouse. Four irradiations, 20 min at a time, were performed at 0.5h, 1.5h, 2.5h, and 3.5h. The *in vivo* fluorescent images of CNC NPs treated mice captured by the IVIS imaging system. **(A)**. The tumor region fluorescent signals dynamic analytics results **(B)**. The data of signal quantitation were extracted at more than three areas. The “***”, “**” and “*” symbols represent P values less than 0.001, 0.01 and 0.05, respectively.

Next, the double-tumor mice were selected to evaluate the triggered release efficacy of CNC NPs *in vivo*. Each mouse only received ultrasound irradiation on its right tumor. **Figure 8A** shows the *in vivo* fluorescence image after ultrasound triggering. For the IR780 injected mouse, the fluorescent signals were strong in the liver and spleen but weak in the tumor. There was a difference between the fluorescent signals of the left and right tumors. The signal of the irradiated tumor (right) was slightly stronger than that of the control tumor (left), indicating that ultrasound irradiation can help drugs enter the tumor tissue. Compared with IR780 injected mouse, CNC NPs injected mouse had stronger fluorescent signals. It is worth noting that the tumor irradiated by the ultrasound showed enhanced fluorescent signals. However, the control tumor was not the case. Quantitative analysis of the fluorescent intensity in the tumor area of two mice further confirmed the advantage of ultrasound triggering (**Figure 8B**).

### Antitumor Effect of CNC NPs *In Vivo*

**Figure 9:**
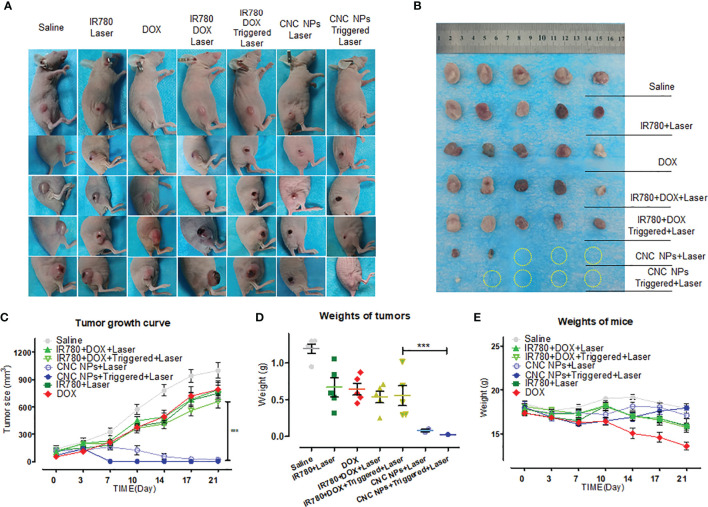
The *in vivo* antitumor effect of CNC NPs. The photographs of the mice in each group during the tumor intervention experiment **(A)**. All of remained tumors after the intervention **(B)**. The tumor growth curve during the tumor intervention experiment (n=5) **(C)**. The tumor weighing results after the intervention (n=5) **(D)**. The mean weight changing curve of the mice during the experiment (n=5) **(E)**. The “***” symbol represents a P values less than 0.001.

**Figure 9A** is a picture of the mice in the tumor intervention experiment. **Figure 9B** is a picture of the tumors taken out after the treatment. Except for the two CNC NPs treated groups, the tumors in the other groups kept growing during the experiment period. Small areas of burn and ulceration were found on the tumor of mice in the free drugs with laser groups, indicating the existence of the PTT effect. However, free drugs with laser combined treatments did not effectively inhibit the tumor growth. The two CNC NPs treated groups showed apparent tumor growth inhibition scenarios, and most of the tumors had been ablated. In the CNC NPs + laser group, there were two tiny tumors with burn scars. After ultrasound triggering, the therapeutic effect of the CNC NPs group was significantly improved, and almost all tumors were ablated. The only remaining tumor was a small one, hidden under the black burn scab on edge. It was found when the scab on the surface was removed after euthanasia where the laser did not reach. The tumor growth curve (**Figure 9C**) and tumor weighing results (**Figure 9D**) further indicate that CNC NPs had an outstanding antitumor effect *in vivo*. These results fully prove that CNC NPs have an excellent therapeutic effect *in vivo*. In addition, during the whole treatment process, the weight of CNC NPs treated mice did not change notably, while the weight of mice in other groups decreased significantly, demonstrating the safety of CNC NPs *in vivo* (**Figure 9E**).

## Discussion

Compared with other kinds of breast cancer, TNBC lacks effective targets for systemic therapy, making it impossible for patients to use other available breast cancer treatment methods. Moreover, in terms of TNBC, the patients are young; the cells have strong invasion capability; the DNA has a high mutation rate; the tumor has a large size and is prone to early recurrence and distant metastasis. It is a kind of primary breast cancer with an inferior prognosis (43, 44). Although there have been many new treatments for TNBC in recent years, their curative effects are still unsatisfactory. Breast cancer is usually not deep in the human body and is suitable for PTT treatments. Research showed that PTT combined with chemotherapy has a synergistic anti-tumor effect on breast cancer (19). However, only when chemotherapeutic drugs and photothermal agents accumulate simultaneously in the tumor site can the synergy be achieved effectively. The molecules have different physical and chemical properties and metabolic pathways *in vivo* and often do not accumulate in tumors simultaneously. Therefore, asynchronous delivery has become the main problem of combination therapy. Different therapeutic molecules can be constructed in one nanocarrier according to the purposes. The synchronous drug delivery to tumor tissue and cells and the synergistic treatment could be realized through targeted delivery and responsive release of the nanocarrier (45). In this study, using PDA as the encapsulation material and PFH as the phase change agent, we realized the efficient synchronous delivery of DOX and IR780 by the ultrasound and pH cascade trigger release technology. PDA plays a crucial role in this drug delivery system. Firstly, PDA is polymerized from dopamine in an alkaline environment. It wrapped the DOX and IR780 *via* π-π stacking, forming a rigid thin shell on the surface of PFH micelle, which ensures the stability of the nanocarrier. On the other hand, because PDA degrades in the acidic environment, CNC NPs can better release drugs in solid tumor tissue, which often has an acidic microenvironment in nature. The rigid shell-core structure of CNC NPs maintains stability of PFH *in vivo*. Then, the ultrasound irradiation to tumor tissue can make the CNC NPs that flowed through or accumulated in the tumor break rapidly to release drugs and increase the absorption of drug molecules there to achieve the effect of synchronous and efficient delivery. All these functions do not require a very complex technique, and the CNC NPs can be prepared by a straightforward method. The results show that the CNC NPs have a shell and a hollow core and have an excellent mono-dispersity. They are particles less than 100 nm in diameter with weak negative charges on their surface and can accumulate rapidly in the tumor through the EPR effect. CNC NPs have good colloidal stability, which effectively prolongs their drug cycle time. Moreover, the nanocarrier has high DOX and IR780 loading capacities. These characteristics indicate that CNC NPs’ application only requires fewer dosing frequency, which reduces the occurrence of side effects and drug resistance.

CNC NPs are expected to enter the systemic circulation through intravenous injection. Therefore, the delivery and release performance are the key to the therapeutic effect. PDA has an outstanding encapsulation performance. It is polymerized by dopamine under alkaline conditions. It can self-polymerize or deposit on various materials. It has high biocompatibility, low toxicity, and pH sensitivity. Many reports about the application of PDA nanocarriers in tumor therapy have been published (46–49). PDA nanocarrier has a super long cycle effect *in vivo* (41). These pieces of evidence show that PDA meets the requirements of CNC NPs. The drug release triggering stimuli are in two categories, internal ones and external ones. The internal stimuli, including pH response, redox, and enzymatic reaction, work inside the tumor tissues and cells, allowing the nanocarriers to degrade and release the loaded drugs slowly. The external stimuli, including the microwave, radiation, and ultrasound, are artificially imposed factors outside the body. The ultrasound as an external stimulus has significant advantages (26, 30). CNC NPs realize the cascade release by combining the internal and external triggering stimuli. The *in vivo* and *in vitro* experiment results have proved that CNC NPs have long circulation ability. The nanocarriers showed excellent colloidal stability in the simulated physiological environment. Only a small amount of loaded molecules were released without triggering. After 144 h *in vivo*, CNC NPs still had a high accumulation in the tumor. This characteristic can be used for monitoring and real-time evaluation of therapeutic effect. The subsequent triggered release results further prove that CNC NPs have excellent triggering and releasing properties. A clinical ultrasound instrument is able to trigger the microbubble burst and drug release rapidly. The *in vivo* experiment results also show that with stimuli, CNC NPs could rapidly release the loaded molecules in the triggered region, causing a significant signal accumulation compared with the non-triggered tumor tissue of the same mouse. This series of results fully prove that CNC NPs have achieved the original design goals of imaging and delivery performance.

As a new treatment *in vivo*, the low-toxicity is crucial to its application. According to CNC NPs’ administration process and mechanism, we first implemented the CCK-8 test and colony formation assay to detect the *in vitro* toxicity of CNC NPs. The results showed that the inhibitory effect of CNC NPs was similar to that of the equivalent concentration DOX, indicating that there was no additional toxicity except for the efficacy of chemotherapy. Then, the hemolysis test, acute toxicity test, histopathological analysis, and inflammatory factor concentration test were utilized to evaluate the safety of CNC NPs *in vivo*. The hemolysis rate was less than 5%, and no mouse died under high-dose injection. No obvious damage was found in pathology and inflammatory factor detection. In general, CNC NPs has good biocompatibility. In terms of tumor treatments, the introduction of PTT can significantly improve the effect of cell ablation *in vitro*, which was stronger than that of chemotherapy or PTT alone. Flow cytometry results also confirmed that CNC NPs caused enhanced cell apoptosis effect. The *in vivo* anti-tumor effect was evaluated in tumor-bearing mice. In the experiment, the dosage was relatively low. Each free drug treatment group did not show significant tumor inhibition. There was no significant difference in tumor size between the free drug group and the negative control group. But the two CNC NPs treatment groups showed an excellent therapeutic effect, and most of the tumors were ablated. The group with ultrasound triggering had significantly better inhibition than that of the group without ultrasound triggering.

## Conclusion

The CNC NPs fully meets original expectations of the study. The NPs can enter the systemic circulation through intravenous injection and maintain a prolonged circulation time *in vivo*. The PFH phase transition of CNC NPs in tumor tissue triggered by ultrasound leads to the breakage of the PDA shell and the release of IR780 and DOX. After that, the remaining PDA is gradually degraded in the acidic environment of tumor tissue and cells, releasing the loaded drug completely. Under laser irradiation, IR780 and residual PDA play the role of PTT agents. IR780 was used as the probe of NIFI and PTT molecule, while DOX can ablate the remaining tumor cells, fully realizing the *in vivo* monitoring and synergistic effect. All the steps were verified by relevant experiments. This study provides experimental evidence for the development of a new treatment for TNBC.

## Data Availability Statement

The raw data supporting the conclusions of this article will be made available by the authors, without undue reservation.

## Ethics Statement

The animal study was reviewed and approved by Laboratory Animal Administration Committee of Xi’an Medical University.

## Author Contributions

KLi, TZ and XL designed the study. KLi, RL, BZ, JC, KLan and DC performed the experiments. KLi, WZ, and TZ analyzed the results and data. KLi, RL, BZ and KLan prepared the manuscript. KLi, TZ and XL modified the manuscript. All authors contributed to the article and approved the submitted version.

## Funding

This study was supported, in part, by the National Natural Science Foundation of China (Program No.81801863), Innovation Capability Support Program of the Shaanxi Province (Program No.2020KJXX-050). The Natural Science Basic Research Plan in Ningxia Province of China (Program No.2021AAC03319). The Research and Development Program of Innovation Chain for Key industries in Shaanxi Province (Program No. 2021ZDLSF02-09). Key Research and Development Program of Shaanxi (Program No.2020NY-118). Research Foundation of China-Nepal Friendship Research Center of Prof. Rajiv Kumar Jha (Program No.18LJM09).

## Conflict of Interest

The authors declare that the research was conducted in the absence of any commercial or financial relationships that could be construed as a potential conflict of interest.

## Publisher’s Note

All claims expressed in this article are solely those of the authors and do not necessarily represent those of their affiliated organizations, or those of the publisher, the editors and the reviewers. Any product that may be evaluated in this article, or claim that may be made by its manufacturer, is not guaranteed or endorsed by the publisher.
